# WALKABLE NEIGHBORHOODS AND BRAIN HEALTH IN LATER LIFE: A SCOPING REVIEW

**DOI:** 10.1093/geroni/igz038.2216

**Published:** 2019-11-08

**Authors:** Boeun Kim

**Affiliations:** University of Washington, Seattle, Washington, United States

## Abstract

Brain health is critical in independent living and well-being in later life. There have been growing interests in investigating the role of walkable neighborhoods in brain health. The purpose of this scoping review was to understand the influence of walkable neighborhoods on cognitive outcomes in older adults. The physical features of walkable neighborhoods and their measurements were examined. Database search identified 2957 peer-reviewed articles and eight studies (2012-2018) met inclusion criteria. Community facilities and land-use mix were consistently associated with cognitive outcomes across studies. The measures of walkable neighborhoods were under development and the measurement properties were not reported in most studies. Further research on the definition of walkable neighborhoods that accounts specifically for older adults with/without cognitive impairment is needed. Additionally, more rigorous, valid, reliable standardized instruments for measuring walkable neighborhoods, as well as a standard for evaluating the quality of instruments are warranted.

